# Association between vitamin A, retinol and carotenoid intake and pancreatic cancer risk: Evidence from epidemiologic studies

**DOI:** 10.1038/srep38936

**Published:** 2016-12-12

**Authors:** Xiaoyi Huang, Yisha Gao, Xiaosong Zhi, Na Ta, Hui Jiang, Jianming Zheng

**Affiliations:** 1 Department of Pathology, Changhai Hospital, Second Military Medical University, Shanghai, P. R. China; 2 Department of Cell Biology, Second Military Medical University, Shanghai, P. R. China

## Abstract

Pancreatic cancer is a devastating disease with poor prognosis. The association between vitamin A, retinol and carotenoid intake and the risk of pancreatic cancer occurrence remains controversial, and therefore it is necessary to make a meta-analysis to clarify the association between vitamin A, retinol and carotenoid intake and pancreatic cancer risk. In the present study, PubMed and EMBASE databases were used to identify qualified studies. The association between dietary vitamin A, retinol and carotenoids was estimated by pooled odds ratios (ORs) and corresponding 95% confidence intervals (CIs). It was found that there was an inverse correlation between vitamin A, beta-carotene and lycopene intake and the risk of pancreatic cancer (for vitamin A, pooled OR = 0.85, 95%CI = 0.74–0.97, P = 0.015; for beta-carotene, pooled OR = 0.78, 95%CI = 0.66–0.92, P = 0.003; for lycopene, pooled OR = 0.84, 95%CI = 0.73–0.97, P = 0.020), which was more prominent in case-control study subgroup. In conclusion, dietary vitamin A, beta-carotene and lycopene might inversely correlate with pancreatic cancer.

Pancreatic cancer is a devastating disease with poor prognosis and the 5-year survival rate remains low at 8%^1^. It is the eighth and ninth leading cause of cancer-related death in men and women respectively throughout the world^2^. For patients with resectable pancreatic cancer, surgery is the mainstay of treatment. But the median overall survival time remains low in all pancreatic cancer stages^3^. There have been few therapeutic advances or effective treatments over the last few years^4^, highlighting the importance of identifying preventive factors for this malignancy. Risk factors such as smoking, obesity, diabetes mellitus, chronic pancreatitis and established genetic syndromes are known to be associated with pancreatic cancer^5^. A number of epidemiologic studies have been published in an attempt to explore the relationship between nutrient intake and the risk of pancreatic cancer occurrence. Various vitamins including vitamin B^6^, vitamin C^7^ and vitamin E^8^ have been implicated in the risk of pancreatic cancer occurrence according to previous studies.

Vitamin A (retinol) and its derivatives are a group of fat soluble compounds composed of a similar structure which are rich in cod liver oil and play important role in multiple biological processes^9^. Due to their ability to promote normal embryonic development and exert effects on cellular differentiation, they are essential for all stages of life from embryogenesis to adulthood^10^. However, they cannot be synthesized de novo by animals (including human) and must be obtained from the diets^11^. Recently, a myriad of epidemiological studies have demonstrated an inverse relationship between dietary vitamin A consumption and cancer development^12^. For instance, vitamin A has been proved to play a protective role in breast cancer^13^ and lung cancer^14^. However, the association between vitamin A (including retinol and carotenoid) and pancreatic cancer remains controversial^15^,^16^,^17^,^18^. Zablotska *et al*. conducted a case–control study to evaluate the association of dietary vitamin D, calcium and retinol and the risk of pancreatic cancer in USA, finding that there was no signification association between them^18^. Also, Kalapothaki *et al*. found that vitamin A intake was not related to pancreatic cancer risk when crude fiber intake was adjusted^16^. The results of clinical studies are not consistent with those of molecular researches. But other carotenoids, such as lycopene, alpha-and beta-carotene, are associated with pancreatic cancer risk^17^,^19^. Therefore, a meta-analysis is necessary to clarify the association between vitamin A, retinol and carotenoids intake and pancreatic cancer risk.

## Results

### Study characteristics and quality assessment

**Figure 1:**
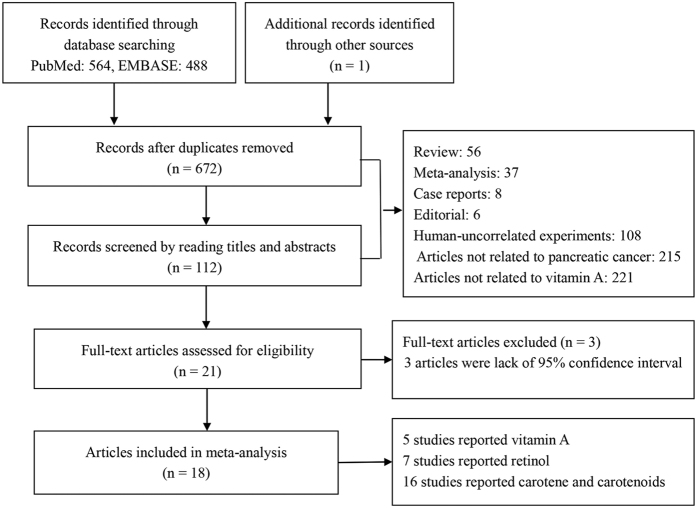
Flow chart of the study selection and inclusion process.

Initially, 672 articles were identified and 18 eligible studies were included in meta-analysis (Fig. 1). The characteristics of the studies and quality assessment results are shown in Table 1. The studies were published from 1990 to 2013. Since the subjects could be divided into males and females, the studies by Ji *et al*., Nkondjock *et al*. and Zablotska *et al*. were separated into two studies, respectively^18^,^19^,^20^. According to different designs of controls, the study conducted by Kalapothaki *et al*. was also divided into two studies^16^. Therefore, there were altogether 22 studies in our meta-analysis, among which 16 were performed in Caucasians, 3 in Asians and 3 in mixed population. Besides, 3 studies were conducted in males only, 3 studies were in females only, and the other 16 in both sexes As for the nutrient type, 6 studies focused on Vitamin A, 11 on retinol, and 17 on carotenoids including 6 on alpha-carotene, 14 on beta-carotene, 8 on lycopene, 6 on crytoxanthin and 7 on lutein and zeaxanthin. Quality assessment was conducted in all included studies, and the Newcastle-Ottawa-Scale (NOS) scores ranged from 6 to 9.

### Quantitative synthesis

#### Vitamin A and pancreatic cancer

**Figure 2:**
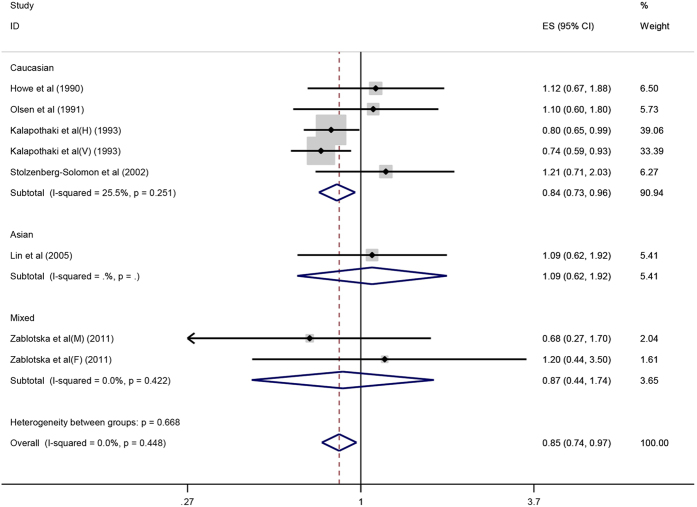
Forest plot of vitamin A intake and pancreatic cancer risk in different populations.

The results pooled by the fixed effect model indicated that there was an inverse association between vitamin A intake and pancreatic cancer risk (OR = 0.85, 95%CI = 0.74–0.97, P = 0.015) (Fig. 2). In addition, stratification analysis conducted by ethnicity and study design type revealed a significant association between vitamin A intake and pancreatic cancer risk in Caucasians subgroup (OR = 0.84, 95%CI = 0.73–0.96, P = 0.011) and case-control subgroup (OR = 0.83, 95%CI = 0.72–0.95, P = 0.007). Subsequently, publication bias was test by funnel plot and Egger’s test. The Egger’s test value suggested that significant publication bias was in the meta-analysis (P = 0.052). The results of metatrim suggested that the summary OR was 0.815 and corresponding 95%CI was 0.702 to 0.946. Besides, no single study could change the results in sensitive analyses, implying that the results of this meta-analysis were robust.

#### Retinol and pancreatic cancer

**Figure 3:**
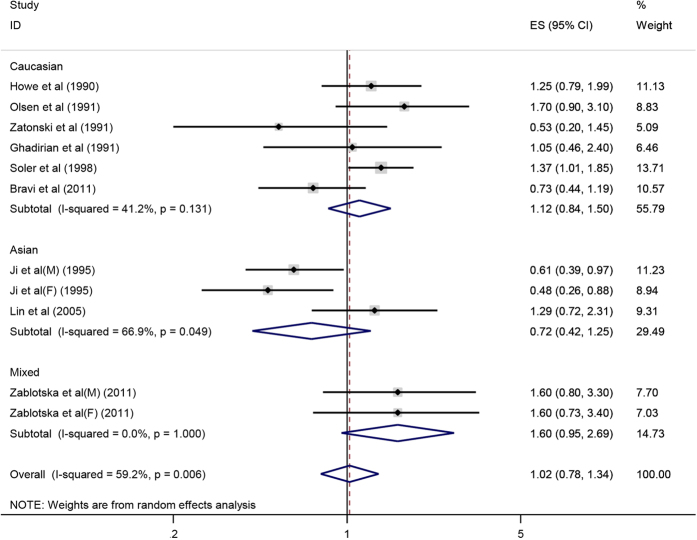
Forest plot of retinol intake and pancreatic cancer risk in different populations.

The meta-analysis based on 11 studies of 9 articles indicated that there was no significant correlation between retinol intake and pancreatic cancer risk (OR = 1.02, 95%CI = 0.78–1.34, P = 0.860). Subgroup analysis by ethnicity and the results showed no significant correlation between retinol intake and the risk of pancreatic cancer (Fig. 3). Additionally, the stability of the results was estimated by sensitive analysis, showing that a good stability and credibility. Publication bias was also tested by funnel plot and Egger’s test (P = 0.591), suggesting that there was no statistically significant publication bias in this meta-analysis.

#### Carotene, alpha-carotene, beta-carotene and pancreatic cancer

**Figure 4:**
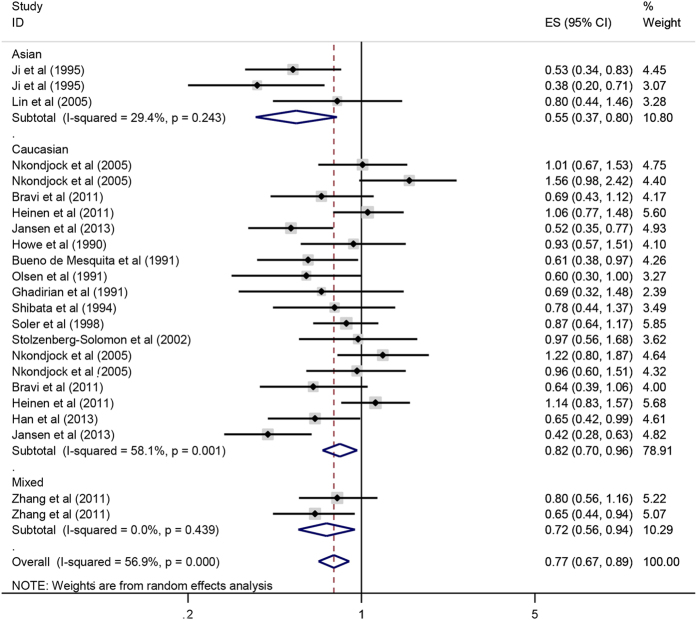
Forest plot of carotene intake and pancreatic cancer risk in different populations.

**Figure 5:**
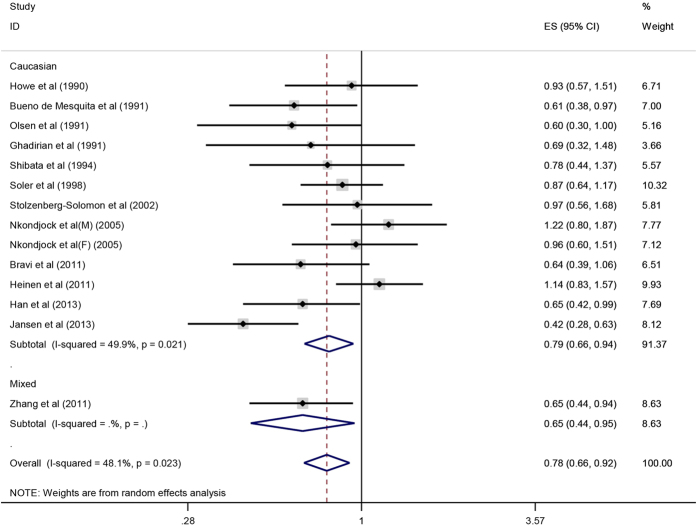
Forest plot of beta-carotene intake and pancreatic cancer risk in different populations.

**Figure 6:**
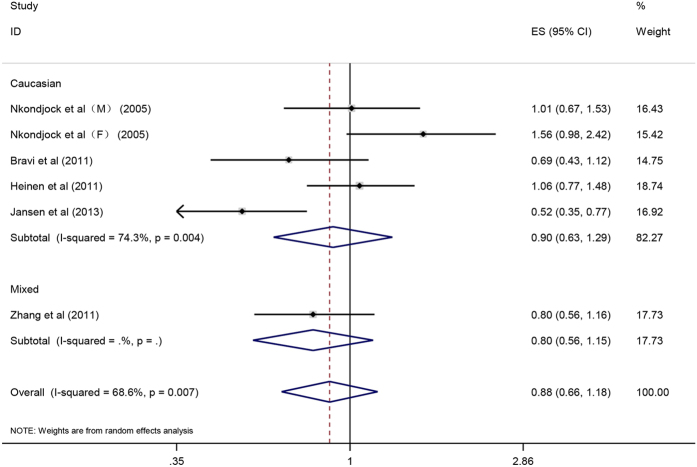
Forest plot of alpha-carotene intake and pancreatic cancer risk in different populations.

**Figure 7:**
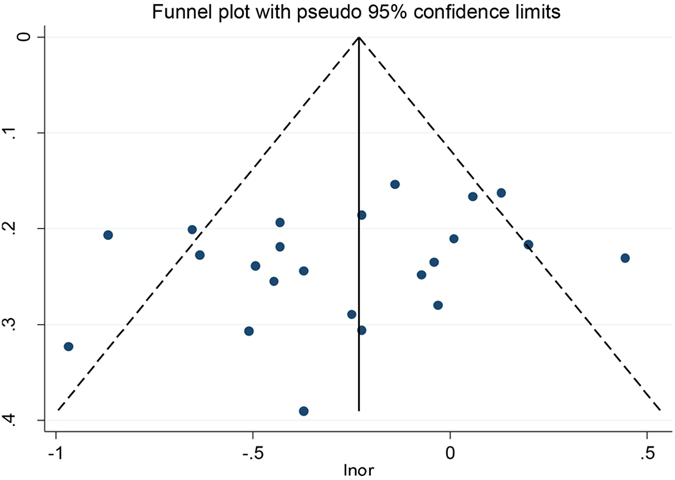
Funnel plot for carotene intake and pancreatic cancer risk.

Overall, there was an inverse correlation between carotene intake and pancreatic cancer risk (OR = 0.77, 95%CI = 0.67–0.89, P < 0.001). After stratification by ethnicity, the association became stronger among Asian population (OR = 0.55, 95%CI = 0.37–0.80, P = 0.002) as compared with that in Caucasian (OR = 0.82, 95%CI = 0.70–0.96, P = 0.016) and the mixed population (OR = 0.72, 95%CI = 0.56–0.94, P = 0.016) (Fig. 4). The result of case-control subgroup showed an inverse association between carotene intake and pancreatic cancer risk (OR = 0.74, 95%CI = 0.62–0.87, P < 0.001), while there was no significant association between carotene intake and pancreatic cancer risk in prospective studies (OR = 0.94, 95%CI = 0.76–1.16, P = 0.577). As for nutrient types, the result showed that there was an inverse correlation between beta-carotene intake and pancreatic cancer risk (OR = 0.78, 95%CI = 0.66–0.92, P = 0.003) (Fig. 5), but no significant correlation was observed between alpha-carotene intake and pancreatic cancer risk (OR = 0.88, 95%CI = 0.66–1.18, P = 0.405) (Fig. 6). Sensitive analysis indicated that the results of this meta-analysis were stable. Publication bias was estimated by forest plot and Egger’s test (P = 0.170) and no significant publication bias was found in carotene meta-analysis (Fig. 7).

#### Lycopene and pancreatic cancer

**Figure 8:**
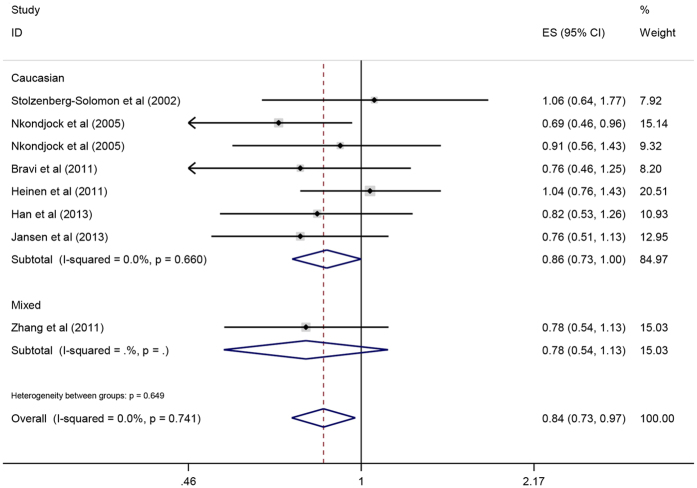
Forest plot of lycopene intake and pancreatic cancer risk in different populations.

As shown in Fig. 8, there was an inverse correlation between lycopene intake and pancreatic cancer risk (OR = 0.84, 95%CI = 0.73–0.97, P = 0.020). When stratified by ethnicity, there was an inverse relationship between lycopene intake and pancreatic cancer risk in Caucasians (OR = 0.86, 95%CI = 0.73–1.00, P = 0.05), while this correlation was insignificant in the mixed population (OR = 0.78, 95%CI = 0.54–1.13, P = 0.187). With respect to the study design type, decreased the pancreatic cancer risk in case-control study (OR = 0.77, 95%CI = 0.64–0.92, P = 0.005), while prospective study showed no association between lycopene intake and pancreatic cancer (OR = 0.98, 95%CI = 0.78–1.23, P = 0.844). The sensitive analysis revealed that the results of this meta-analysis were credible and stable. Publication bias was evaluated by forest plot and Egger’s test (P = 0.857), and the results showed no significant publication bias in the meta-analysis.

#### Cryptoxanthin and pancreatic cancer

The results showed no significant association between cryptoxanthin intake and pancreatic cancer risk (OR = 0.86, 95%CI = 0.67–1.12, P = 0.276). After stratification by ethnicity, there was no significant association in Caucasians and the mixed population. Similarly, there was no significant correlation in Caucasians and the mixed population. Subgroup analysis showed no significant correlation between cryptoxanthin intake and pancreatic cancer risk in case-control study and prospective study. The sensitive analysis implied that the result of the meta-analysis was robustness. The forest plot and Egger’s test (P = 0.522) suggested that no significant publication bias in this meta-analysis.

#### Lutein and zeaxanthin and pancreatic cancer

**Figure 9:**
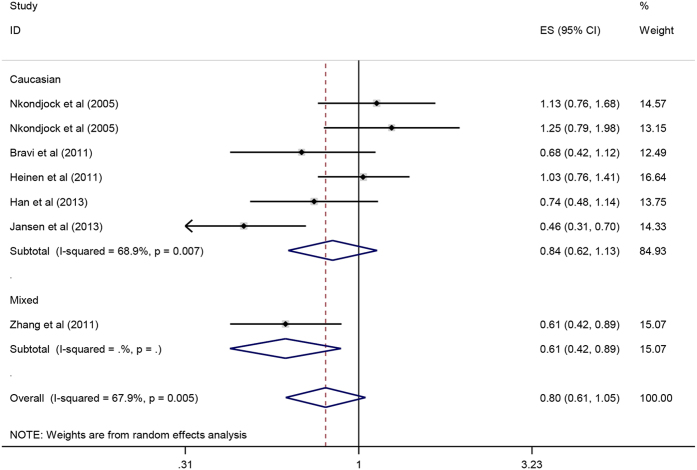
Forest plot of lutein and zeaxanthin intake and pancreatic cancer risk in different populations.

Pooled ORs and corresponding 95%CIs indicated that there was no significant correlation between lutein and zeaxanthin intake and pancreatic cancer risk (OR = 0.80, 95%CI = 0.61–1.05, P = 0.104). As it showed in Fig. 9, there was an inverse association between lutein and zeaxanthin intake and pancreatic cancer risk in the mixed population (OR = 0.61, 95%CI = 0.42–0.89, P = 0.010), but not in Caucasians population (OR = 0.84, 95%CI = 0.62–1.3, P = 0.251). Besides, subgroup analysis showed no significant association could be found in the subgroup of case-control study and prospective study. The sensitive analysis implied that this result was robust. Furthermore, the forest plot and Egger’s test (P = 0.664) showed no significant publication bias in our meta-analysis.

In summary, there was an inverse correlation between vitamin A (including some carotenoids) intake and pancreatic cancer risk, but no significant correlation was observed between retinol intake and pancreatic cancer risk. All the results were summarized in Table 2.

## Discussion

This meta-analysis included 18 articles focusing on the correlation between vitamin A, retinol and carotenoid intake and pancreatic cancer risk. The result showed that dietary vitamin A, carotene, beta-carotene and lycopene were inversely correlated with the risk of pancreatic cancer risk. However, retinol, alpha-carotene, cryptoxanthin, lutein and zeaxanthin intake had no relationship with pancreatic cancer risk.

Vitamin A is a necessity for cell growth and differentiation of epithelial tissues and must be obtained from diets in the human body. Provitamin A compounds, such as beta-carotene can transform into vitamin A, which is an essential molecule entailing multiple developmental pathways and influencing cell proliferation and differentiation in a variety of cell types^21^. Molecular studies had demonstrated that retinoids (vitamin A and its metabolites) could cause apoptosis in pancreatic cancer cells and thus suppress pancreatic cancer growth via activation of retinoic acid receptor-gamma, suggesting that vitamin A and its metabolites may play a protective role against pancreatic cancer^22^. Additionally, several preclinical studies showed that retinols play roles in many signaling pathways related with cell growth, adhesion and migration^23^,^24^,^25^. A recent study revealed that retinoic acid could inhibit pancreatic cancer cell migration and epithelial-mesenchymal transition by decreasing the expression of interleukin 6 (IL-6) in cancer-associated fibroblast (CAFs) cells^25^, suggesting that retinoids could be applied for prevention or therapy the recurrence and metastasis of pancreatic cancer. Actually, immunotherapy including 13-cis-retinoic acid and interleukin 2 had been used for treating locally advanced pancreatic cancer^26^. However, there is no clinical study focusing on vitamin A therapy in pancreatic cancer so far.

Other carotenoids such as lycopene and zeaxanthin that cannot convert into vitamin A may act as antioxidants against cancer initiation and progression. Lycopene has been proved to be a potent inhibitor for cell proliferation and growth in some cancer cells, such as endometrial cancer, breast cancer and lung cancer^27^,^28^. More recently, Assar and colleagues reported that lycopene could suppress the nuclear factor kappa B (NF-κB) signaling pathway through inhibiting phosphorylation of inhibitor of kappa B (IκB) in human prostate and breast cancer cells, probably due to the action of lycopene as an antioxidant to scavenge free radicals^29^. These data provide a potential strategy to prevent and treat pancreatic cancer by using lycopene.

Several meta-analyses or pooled analyses have investigated the association between the intake of other vitamins and pancreatic cancer risk. Fan *et al*. conducted a meta-analysis to assess the relationship between dietary vitamin C and pancreatic cancer risk and found that a higher vitamin C intake was inversely correlated with pancreatic cancer risk^7^. Similarly, dietary vitamin E was found to be a protective factor against pancreatic cancer^8^. However, a recent pooled analysis suggested that higher levels of vitamin D might increase pancreatic cancer risk^30^. To the best of our knowledge, this is the first meta-analysis about the relationship of dietary vitamin A, retinol, and carotenoids with pancreatic cancer risk. Overall, we found that dietary vitamin A had an inversely association with pancreatic cancer risk. On the contrary, several studies had investigated the relationship between vitamin A intake and the risk of pancreatic cancer and the results were negative. Partly, these conclusions may due to the small sample size of each study. Besides, the only prospective study showed no association between vitamin A intake and the risk of pancreatic cancer^31^. However, this prospective study was based on male smokers instead of the general population. No significant relationship was found between dietary retinol and pancreatic cancer risk, or in the subgroups of Caucasians, Asians and the mixed population. These results are consistent with previous case-control studies^15^,^17^,^32^,^33^,^34^,^35^.

The association between carotenoid intake and cancer risk had been investigated in many cancer types. Zhou *et al*. conducted a meta-analysis and found that beta-carotene and alpha-carotene were inversely correlated with risk of gastric cancer^36^. Another meta-analysis revealed that dietary alpha-carotene and lycopene could decrease the risk of prostate cancer^37^. In our meta-analysis, beta-carotene and lycopene intake were inversely associated with pancreatic cancer risk, while alpha-carotene and cryptoxanthin intake had no significant relationship with pancreatic cancer risk. Many previous observational studies including 10 case-control studies^15^,^17^,^19^,^32^,^33^,^38^,^39^,^40^,^41^ and 4 prospective studies^31^,^42^,^43^,^44^ on the relationship between beta-carotene intake and pancreatic cancer risk reported inconsistent results. These discrepant results from case-control studies might result from recall bias of self-reported dietary intake and different ethnicity. However, the results of 4 prospective studies did not suggest that beta-carotene acted as a protective factor against pancreatic cancer. Notably, a nested case–control study performed in Europe indicated that higher plasma concentrations of beta-carotene could decrease the risk of suffering from pancreatic cancer^45^. Besides, this article also suggested that higher plasma concentrations of zeaxanthin might be inversely related to pancreatic cancer risk, which is consistent with the result of our meta-analysis indicated that the relationship between the total lutein and zeaxanthin intake and pancreatic cancer risk in the subgroup of the mixed population. However, this subgroup result was based on only one case-control study^38^.

Between-study heterogeneity is common in meta-analysis^46^, and the heterogeneity test showed moderate between-study heterogeneity in most meta-analyses. The study characteristics of each study may lead to heterogeneity. In our meta-analysis, the study design, geographic location, publication year and sources of control and cases are various. To find the causes of heterogeneity for covariates, we conducted a meta-regression and subgroup analysis. As a result, meta-regression failed to determine any study characteristics including publication year, study type, study size and ethnicity as sources of heterogeneity. Subgroup analyses by study type and ethnicity were performed to explore the source of heterogeneity. However, between-study heterogeneity was permanent in some subgroups, suggesting that other unknown confounding factors may be present. In addition, the adjustments and the intake levels of nutrients are different between these studies.

Although the results obtained in our meta-analysis are statistically significant as a whole, several limitations should be noted in interpreting our study. First, most included studies in our meta-analysis were case-control studies, in which recall bias may be unavoidable. Both case-control and cohort study are observational study, they require fewer resources but provide less evidence compared with randomized controlled trial (RCT). However, given the extremely low morbidity of pancreatic cancer, there is too difficult to conduct RCT on the association between vitamin A, retinol and carotenoid intake and the risk of pancreatic cancer. Second, some confounding factors such as eating habits and residual confounding cannot be measured, which may affect the stability and credibility of our meta-analysis. Third, the individual sample sizes for each case in most studies included in this meta-analysis were relatively small and these studies were conducted in different populations whose heredity might be different. Finally, some pancreatic cancer cases may be familial heredity^47^, which may change the morbidity of pancreatic cancer and lead to an inaccurate results in epidemiological study.

In conclusion, the results of our meta-analysis indicate that high-level vitamin A, carotene, beta-carotene and lycopene intake might be the potential factors related to low pancreatic cancer risk. However, due to the limitations of the present meta-analysis mentioned above, it should be prudent to make recommendations based on the results of the present meta-analysis.

## Materials and Methods

### Literature search strategy

PubMed and EMBASE databases were used to identify observational studies that reported the association between vitamin A, retinol and carotenoid intake and the risk of PANCREATIC CANCER up to December 30^th^, 2015 by using the following key words “Vitamin A or Vitamin or diet or dietary or retinol or carotenoids or carotene or cryptoxanthin or lycopene or lutein or zeaxanthin” and “pancreatic” and “cancer or carcinoma or neoplasm or tumor or adenocarcinoma”. Additionally, some potential studies were identified via secondary searches which were conducted by searching reference lists of selected literatures.

### Inclusion and exclusion criteria

The inclusion criteria were: (1) observational studies including case-control and cohort study design; (2) studies reporting the association between exposure factors including vitamin A, retinol and carotenoids and the risk of pancreatic cancer; (3) studies published in English or Chinese; (4) Providing the odds ratio (OR) (or relative risk [RR], hazard risk [HR]) data and the corresponding 95% corresponding interval (CI) for the highest vs. the lowest level of vitamin A intake or retinol intake or other carotenoid intake. The exclusion criteria were: (1) reviews, meta-analyses, case reports, editorials or human-uncorrelated experiments; (2) duplicated study (If duplicated studies were present, the study with the largest sample size was selected); (3) studies not reporting OR(or RR, HR) and 95%CI or lacking sufficient data to calculate OR(or RR and HR) and 95%CI.

### Data extraction

The process of data extraction was conducted by two authors independently with a standardized form based on the inclusion and exclusion criteria mentioned above. Any divergence was resolved by rechecking until consensus was reached. The following information was collected: the last name of the first author, publication year, country, ethnicity, study design, number of cases and controls or total sample size, carotenoid types, OR(or RR, HR), the corresponding 95% CI from the most fully adjusted model for the highest vs. the lowest vitamin A intake, and the factors of adjustment for covariates.

### Quality assessment

Newcastle-Ottawa-Scale (NOS) was applied in quality assessment^48^. During this process, the quality of the selected observational studies was evaluated independently by two authors. The NOS is a nine-point scale containing three parts: selection (four points), comparability (two points) and exposure/outcome assessment (three points). A study with a NOS score ≥6 was regarded as a high-quality study, and vice versa.

### Statistical analysis

If the outcome under study is rare in all populations and subgroups under review, one can generally ignore the distinctions between the various measures of relative risk^49^. Given the low absolute risk of pancreatic cancer in the general populations, we interpreted all risk estimates as OR for simplicity. The relationship between vitamin A, retinol and other carotenoids and the risk of pancreatic cancer was assessed by calculating pooled OR and 95%CI respectively. Additionally, subgroup analysis was conducted by study design type and ethnicity if sufficient data was provided. All the statistical tests were two-sided and the results were considered as statistically significant if P ≤ 0.05. The heterogeneity test was performed by Q test and *I*^*2*^. When *I*^*2*^ > 50%, the random effect model was suggested to calculate the pooled OR and 95%CI. Otherwise, the fixed effect model was applied. Furthermore, sensitivity analysis was conducted by omitting each study once a time. We also assessed publication bias via funnel plots and Egger’s test^50^. This meta-analysis was performed by STATA12.0 (STATA Corporation, College Station, TX).

## Additional Information

**How to cite this article**: Huang, X. *et al*. Association between vitamin A, retinol and carotenoid intake and pancreatic cancer risk: Evidence from epidemiologic studies. *Sci. Rep.* **6**, 38936; doi: 10.1038/srep38936 (2016).

**Publisher's note:** Springer Nature remains neutral with regard to jurisdictional claims in published maps and institutional affiliations.

## Figures and Tables

**Table 1:** Characteristics of the studies of vitamin A, retinol and carotenoids intake and pancreatic cancer risk included in this analysis.

Author	Year	Ethnicity	Study design	Country	Sample size (cases/control)	Carotenoid types	OR(RR, HR)*	95%CI	Adjustment for covariates	Newcastle- Ottawa Score
Howe *et al*.^15^	1990	Caucasian	Case-control	Canada	249/505	Vitamin A	RR 1.12	0.67–1.88	None	7
						Retinol	RR 1.25	0.79–1.99		
						Beta-carotene	RR 0.93	0.57–1.51		
Bueno de Mesquita *et al*.^39^	1991	Caucasian	Case-control	Netherlands	164/480	Beta-carotene	OR 0.61	0.38–0.97	Adjusted for age, gender, response status, total smoking and dietary intake of energy	7
Olsen *et al*.^32^	1991	Caucasian	Case-control	USA	212/220	Vitamin A	OR 1.10	0.60–1.80	Adjusted for total energy, energy- adjusted total fat, age, cigarette usage, alcohol consumption, respondent- reported history of diabetes mellitus, and educational level	8
						Retinol	OR 1.70	0.90–3.10		
						Beta-carotene	OR 0.60	0.30–1.00		
Zatonski *et al*.^34^	1991	Caucasian	Case-control	Poland	110/195	Retinol	RR 0.53	0.20–1.45	Adjusted for cigarette lifetime consumption and calories	8
Ghadirian *et al*.^33^	1991	Caucasian	Case-control	Canada	179/239	Retinol	OR 1.05	0.46–2.40	Adjusted for age, sex, lifetime cigarette consumption, response status and energy	8
						Beta-carotene	OR 0.69	0.32–1.48		
Kalapothaki *et al*.^16^	1993	Caucasian	Case-control (hospital control)	Greece	181/181	Vitamin A	OR 0.80	0.65–0.99	Adjusted for age, gender, hospital, past residence, years of schooling, cigarette smoking, diabetes mellitus and energy intake	6
Kalapothaki *et al*.^16^	1993	Caucasian	Case-control (visitor control)	Greece	181/181	Vitamin A	OR 0.74	0.59–0.93	Adjusted for age, gender, hospital, past residence, years of schooling, cigarette smoking, diabetes mellitus and energy intake	7
Shibata *et al*.^42^	1993	Caucasian	Prospective	USA	63/13976	Beta-carotene	RR 0.78	0.44–1.37	Adjusted for sex, age and cigarette smoking	7
Ji *et al*.^20^	1995	Asian (male)	Case-control	China	261/847	Carotene	OR 0.53	0.34–0.83	Adjusted for age, income, smoking, response status and total calories	8
						Retinol	OR 0.61	0.39–0.97		
Ji *et al*.^20^	1995	Asian (female)	Case-control	China	184/680	Carotene	OR 0.38	0.20–0.71	Adjusted for age, income, smoking, green tea drinking, response status and total calories	8
						Retinol	OR 0.48	0.26–0.88		
Soler *et al*.^40^	1998	Caucasian	Case-control	Italy	362/1552	Beta-carotene	OR 0.87	0.64–1.17	Adjusted for age, sex, education, tobacco consumption and area of residence	6
						Retinol	OR 1.37	1.01–1.85		
Stolzenberg-Solomon *et al*.^31^	2002	Caucasian	Prospective	Finland	163/26948	Vitamin A	HR 1.21	0.71–2.03	Adjusted for energy intake, energy-adjusted folate intake	8
						Carotenoids	HR 0.88	0.50–1.55		
						Beta-carotene	HR 0.97	0.56–1.68		
						Lycopene	HR 1.06	0.64–1.77		
Nkondjock *et al*.^19^	2005	Caucasian (male)	Case-control	Canada	258/2331	Alpha-carotene	OR 1.01	0.67–1.53	Adjusted for age, province, smoking, educational attainment, BMI, folate, and total energy intake	7
						Beta-carotene	OR 1.22	0.80–1.87		
						P-cryptoxanthin	OR 1.09	0.72–1.62		
						Lycopene	OR 0.69	0.46–0.96		
						Lutein + Zeaxanthin	OR 1.13	0.76–1.68		
						Total carotenoids	OR 1.22	0.80–1.86		
Nkondjock *et al*.^19^	2005	Caucasian (female)	Case-control	Canada	204/2390	Alpha-carotene	OR 1.56	0.98–2.42	Adjusted for age, province, smoking, educational attainment, BMI, folate, and total energy intake	7
						Beta-carotene	OR 0.96	0.60–1.51		
						P-cryptoxanthin	OR 1.23	0.80–1.87		
						Lycopene	OR 0.91	0.56–1.43		
						Lutein + Zeaxanthin	OR 1.25	0.79–1.98		
						Total carotenoids	OR 0.91	0.58–1.44		
Lin *et al*.^35^	2005	Asian	Case-control	Japan	109/218	Vitamin A	OR 1.09	0.62–1.92	Adjusted for energy intake, age and pack-years of smoking	7
						Retinol	OR 1.29	0.72–2.31		
						Carotene	OR 0.80	0.44–1.46		
Bravi *et al*.^17^	2011	Caucasian	Case-control	Italy	326/652	Retinol	OR 0.73	0.44–1.19	Adjusted for year of interview, education, tobacco smoking, history of diabetes, body mass index, and total energy intake	7
						Alpha-carotene	OR 0.69	0.43–1.12		
						Beta-carotene	OR 0.64	0.39–1.06		
						Beta-cryptoxanthin	OR 0.66	0.39–1.09		
						Lycopene	OR 0.76	0.46–1.25		
						Lutein + Zeaxanthin	OR 0.68	0.42–1.12		
						Total carotenoids	OR 0.64	0.40–1.03		
Zhang *et al*.^38^	2011	Mixed	Case-control	USA	150/459	Lutein + Zeaxanthin	OR 0.61	0.42–0.89	Adjusted for energy intake, body mass index, race, education, smoking, history of diabetes, physical activity, alcohol consumption and energy intake by the residual method	7
						Beta-cryptoxanthin	OR 0.89	0.61–1.28		
						Lycopene	OR 0.78	0.54–1.13		
						Alpha-carotene	OR 0.80	0.56–1.16		
						Beta-carotene	OR 0.65	0.44–0.94		
Zablotska *et al*.^18^	2011	Mixed (male)	Case-control	USA	167/466	Vitamin A	OR 0.68	0.27–1.70	Adjusted for energy intake, body mass index, race, education, smoking, history of diabetes, physical activity, alcohol consumption and energy intake by the residual method	8
					171/490	Retinol	OR 1.60	0.80–3.30		
Zablotska *et al*.^18^	2011	Mixed (female)	Case-control	USA	124/382	Vitamin A	OR 1.20	0.44–3.50	Adjusted for energy intake, body mass index, race, education, smoking, history of diabetes, physical activity, alcohol consumption and energy intake by the residual method	8
					131/407	Retinol	OR 1.60	0.73–3.40		
Heinen *et al*.^43^	2011	Caucasian	Prospective	Netherlands	423/3868	Alpha-carotene	HR 1.06	0.77–1.48	Adjusted for age and sex.	8
						Beta-carotene	HR 1.14	0.83–1.57		
						Lutein + Zeaxanthin	HR 1.03	0.76–1.41		
						Beta-cryptoxanthin	HR 0.77	0.56–1.07		
						Lycopene	HR 1.04	0.76–1.43		
Han *et al*.^44^	2011	Caucasian	Prospective	USA	162/70332	Beta-carotene	HR 0.65	0.42–0.99	Adjusted for age, gender, ethnicity, education, body mass index, physical activity, cigarette smoking status, total alcohol consumption, family history of and total energy intake, pancreatic cancer, history of diabetes	9
						Lutein + Zeaxanthin	HR 0.74	0.48–1.14		
						Lycopene	HR 0.82	0.53–1.26		
Jansen *et al*.^41^	2013	Caucasian	Case-control	USA	384/983	Alpha-carotene	OR 0.52	0.35–0.77	Adjusted for energy, smoking, BMI, age, sex, and drinks of alcohol per week	6
						Beta-carotene	OR 0.42	0.28–0.63		
						Beta-cryptoxanthin	OR 0.55	0.37–0.82		
						Lutein + Zeaxanthin	OR 0.46	0.31–0.70		
						Lycopene	OR 0.76	0.51–1.13		

^*^OR: Odds ratio; RR: risk ratio; HR: hazard ratio.

**Table 2:** The results of the association between vitamin A, retinol and carotenoids intake and the risk of pancreatic cancer in meta-analysis.

	Pooled OR	95%CI	P_a_	Model	*I*^*2*^	P_b_	The number of studies
Vitamin A							
Overall	0.85	0.74–0.97	0.015	Fixed	0.0	0.448	8
Subgroup							
Caucasian	0.84	0.73–0.96	0.011	Fixed	25.5	0.251	5
Asian	1.09	0.62–1.92	0.765	Fixed	—	—	1
Mixed	0.87	0.44–1.74	0.700	Fixed	0.0	0.422	2
Case-control	0.83	0.72–0.95	0.007	Fixed	0.0	0.549	7
Prospective	1.21	0.72–2.05	0.477	Fixed	—	—	1
Retinol							
Overall	1.02	0.78–1.34	0.860	Random	59.2	0.006	11
Subgroup							
Caucasian	1.12	0.84–1.50	0.437	Random	41.2	0.131	6
Asian	0.72	0.42–1.25	0.240	Random	66.9	0.049	3
Mixed	1.60	0.95–2.69	0.077	Random	0.0	1	2
Carotenoids							
Carotene							
Overall	0.77	0.67–0.89	<0.001	Random	56.9	<0.001	23
Subgroup							
Caucasian	0.82	0.70–0.96	0.016	Random	58.1	0.001	18
Asian	0.55	0.37–0.80	0.002	Random	29.4	0.243	3
Mixed	0.72	0.56–0.94	0.016	Random	0.0	0.439	2
Case-control	0.74	0.62–0.87	<0.001	Random	57.9	0.001	18
Prospective	0.94	0.76–1.16	0.577	Random	56.9	<0.001	5
Alpha-carotene							
Overall	0.88	0.66–1.18	0.405	Random	68.6	0.007	6
Subgroup							
Caucasian	0.90	0.63–1.29	0.577	Random	74.3	0.004	5
Mixed	0.80	0.56–1.15	0.230	Random	—	—	1
Case-control	0.85	0.60–1.21	0.362	Random	72.2	0.006	5
Prospective	1.06	0.761.47	0.727	Random	—	—	1
Beta-carotene							
Overall	0.78	0.66–0.92	0.003	Random	48.1	0.023	14
Subgroup							
Caucasian	0.79	0.66–0.94	0.009	Random	49.9	0.021	13
Mixed	0.65	0.44–0.95	0.026	Random	—	—	1
Case-control	0.74	0.60–0.90	0.003	Random	49.8	0.036	10
Prospective	0.89	0.67–1.18	0.414	Random	35.0	0.202	4
Lycopene							
Overall	0.84	0.73–0.97	0.020	Fixed	0.0	0.741	8
Subgroup							
Caucasian	0.86	0.73–1.00	0.050	Fixed	0.0	0.660	7
Mixed	0.78	0.54–1.13	0.187	Fixed	—	—	1
Case-control	0.77	0.64–0.92	0.005	Fixed	0.0	0.933	5
Prospective	0.98	0.78–1.23	0.844	Fixed	0.0	0.645	3
Cryptoxanthin							
Overall	0.86	0.67–1.12	0.276	Random	57.3	0.0507	5
Subgroup							
Caucasian	0.86	0.61–1.21	0.387	Random	67.8	0.0811	4
Mixed	0.89	0.61–1.29	0.538	Random	—	—	1
Case-control	0.90	0.64–1.26	0.530	Random	66.0	0.0802	4
Prospective	0.77	0.56–1.07	0.114	Random	—	—	1
Lutein + zeaxanthin							
Overall	0.80	0.61–1.05	0.104	Random	67.9	0.005	7
Subgroup							
Caucasian	0.84	0.62–1.13	0.251	Random	68.9	0.007	6
Mixed	0.61	0.42–0.89	0.010	Random	—	—	1
Case-control	0.77	0.53–1.11	0.163	Random	74.5	0.003	5
Prospective	0.91	0.66–1.24	0.537	Random	32.7	0.223	2

P_a_: P value for meta-analysis; P_b_: P value for heterogeneity test.

## References

[b1] Siegel R. L., Miller K. D. & Jemal A. Cancer statistics. CA: a cancer journal for clinicians 66, 7–30, doi: 10.3322/caac.21332 (2016). 26742998

[b2] Ryan D. P., Hong T. S. & Bardeesy N. Pancreatic adenocarcinoma. The New England journal of medicine 371, 1039–1049, doi: 10.1056/NEJMra1404198 (2014). 25207767

[b3] Hidalgo M. Pancreatic cancer. The New England journal of medicine 362, 1605–1617, doi: 10.1056/NEJMra0901557 (2010). 20427809

[b4] Sharma J., Duque M. & Saif M. W. Emerging therapies and latest development in the treatment of unresectable pancreatic neuroendocrine tumors: an update for clinicians. Therapeutic advances in gastroenterology 6, 474–490, doi: 10.1177/1756283x13498808 (2013). 24179483 PMC3808571

[b5] Connor A. A. & Gallinger S. Hereditary Pancreatic Cancer Syndromes. Surgical oncology clinics of North America 24, 733–764, doi: 10.1016/j.soc.2015.06.007 (2015). 26363539

[b6] Gong Z., Holly E. A. & Bracci P. M. Intake of folate, vitamins B6, B12 and methionine and risk of pancreatic cancer in a large population-based case-control study. Cancer causes & control: CCC 20, 1317–1325, doi: 10.1007/s10552-009-9352-9 (2009). 19415507 PMC3306816

[b7] Fan H. . Association between vitamin C intake and the risk of pancreatic cancer: a meta-analysis of observational studies. Scientific reports 5, 13973, doi: 10.1038/srep13973 (2015). 26360104 PMC4566085

[b8] Peng L., Liu X., Lu Q., Tang T. & Yang Z. Vitamin E intake and pancreatic cancer risk: a meta-analysis of observational studies. Medical science monitor: international medical journal of experimental and clinical research 21, 1249–1255, doi: 10.12659/msm.893792 (2015). 25929754 PMC4428318

[b9] Das B. C. . Retinoic acid signaling pathways in development and diseases. Bioorganic & medicinal chemistry 22, 673–683, doi: 10.1016/j.bmc.2013.11.025 (2014). 24393720 PMC4447240

[b10] Rochette-Egly C. Retinoic acid signaling and mouse embryonic stem cell differentiation: Cross talk between genomic and non-genomic effects of RA. Biochimica et biophysica acta 1851, 66–75, doi: 10.1016/j.bbalip.2014.04.003 (2015). 24768681

[b11] Harrison E. H. Mechanisms involved in the intestinal absorption of dietary vitamin A and provitamin A carotenoids. Biochimica et biophysica acta 1821, 70–77, doi: 10.1016/j.bbalip.2011.06.002 (2012). 21718801 PMC3525326

[b12] Doldo E., Costanza G. & Agostinelli S. Vitamin A, cancer treatment and prevention: the new role of cellular retinol binding proteins. 2015, 624627, doi: 10.1155/2015/624627 (2015). PMC4387950 25879031

[b13] Fulan H. . Retinol, vitamins A, C, and E and breast cancer risk: a meta-analysis and meta-regression. Cancer causes & control: CCC 22, 1383–1396, doi: 10.1007/s10552-011-9811-y (2011). 21761132

[b14] Chen G., Wang J., Hong X., Chai Z. & Li Q. Dietary vitamin E intake could reduce the risk of lung cancer: evidence from a meta-analysis. International journal of clinical and experimental medicine 8, 6631–6637 (2015). 26131295 PMC4483938

[b15] Howe G. R., Jain M. & Miller A. B. Dietary factors and risk of pancreatic cancer: results of a Canadian population-based case-control study. International journal of cancer. Journal international du cancer 45, 604–608 (1990). 2157670 10.1002/ijc.2910450405

[b16] Kalapothaki V. . Nutrient intake and cancer of the pancreas: a case-control study in Athens, Greece. Cancer causes & control: CCC 4, 383–389 (1993). 8394150 10.1007/BF00051342

[b17] Bravi F. . Dietary intake of selected micronutrients and the risk of pancreatic cancer: an Italian case-control study. Annals of oncology: official journal of the European Society for Medical Oncology/ESMO 22, 202–206, doi: 10.1093/annonc/mdq302 (2011). 20530201

[b18] Zablotska L. B., Gong Z., Wang F., Holly E. A. & Bracci P. M. Vitamin D, calcium, and retinol intake, and pancreatic cancer in a population-based case-control study in the San Francisco Bay area. Cancer causes & control: CCC 22, 91–100, doi: 10.1007/s10552-010-9678-3 (2011). 21072578 PMC3002162

[b19] Nkondjock A., Ghadirian P., Johnson K. C. & Krewski D. Dietary intake of lycopene is associated with reduced pancreatic cancer risk. The Journal of nutrition 135, 592–597 (2005). 15735099 10.1093/jn/135.3.592

[b20] Ji B. T. . Dietary factors and the risk of pancreatic cancer: a case-control study in Shanghai China. Cancer epidemiology, biomarkers & prevention: a publication of the American Association for Cancer Research, cosponsored by the American Society of Preventive Oncology 4, 885–893 (1995). 8634662

[b21] Mongan N. P. & Gudas L. J. Diverse actions of retinoid receptors in cancer prevention and treatment. Differentiation; research in biological diversity 75, 853–870, doi: 10.1111/j.1432-0436.2007.00206.x (2007). 17634071

[b22] Pettersson F., Dalgleish A. G., Bissonnette R. P. & Colston K. W. Retinoids cause apoptosis in pancreatic cancer cells via activation of RAR-gamma and altered expression of Bcl-2/Bax. British journal of cancer 87, 555–561, doi: 10.1038/sj.bjc.6600496 (2002). 12189556 PMC2376147

[b23] Rosewicz S., Brembeck F., Kaiser A., Marschall Z. V. & Riecken E. O. Differential growth regulation by all-trans retinoic acid is determined by protein kinase C alpha in human pancreatic carcinoma cells. Endocrinology 137, 3340–3347, doi: 10.1210/endo.137.8.8754760 (1996). 8754760

[b24] Rosewicz S. . Retinoids inhibit adhesion to laminin in human pancreatic carcinoma cells via the alpha 6 beta 1-integrin receptor. Gastroenterology 112, 532–542 (1997). 9024307 10.1053/gast.1997.v112.pm9024307

[b25] Guan J. . Retinoic acid inhibits pancreatic cancer cell migration and EMT through the downregulation of IL-6 in cancer associated fibroblast cells. Cancer letters 345, 132–139, doi: 10.1016/j.canlet.2013.12.006 (2014). 24334138

[b26] Recchia F. . Chemoradioimmunotherapy in locally advanced pancreatic and biliary tree adenocarcinoma: a multicenter phase II study. Pancreas 38, e163–168, doi: 10.1097/MPA.0b013e3181abe222 (2009). 19531969

[b27] Levy J. . Lycopene is a more potent inhibitor of human cancer cell proliferation than either alpha-carotene or beta-carotene. Nutrition and cancer 24, 257–266, doi: 10.1080/01635589509514415 (1995). 8610045

[b28] Tang L., Jin T., Zeng X. & Wang J. S. Lycopene inhibits the growth of human androgen-independent prostate cancer cells *in vitro* and in BALB/c nude mice. The Journal of nutrition 135, 287–290 (2005). 15671228 10.1093/jn/135.2.287

[b29] Assar E. A., Vidalle M. C., Chopra M. & Hafizi S. Lycopene acts through inhibition of IkappaB kinase to suppress NF-kappaB signaling in human prostate and breast cancer cells. Tumour biology: the journal of the International Society for Oncodevelopmental Biology and Medicine, doi: 10.1007/s13277-016-4798-3 (2016). 26779636

[b30] Waterhouse M. . Vitamin D and pancreatic cancer: a pooled analysis from the Pancreatic Cancer Case-Control Consortium. Annals of oncology: official journal of the European Society for Medical Oncology/ESMO 26, 1776–1783, doi: 10.1093/annonc/mdv236 (2015). PMC4511221 25977560

[b31] Stolzenberg-Solomon R. Z., Pietinen P., Taylor P. R., Virtamo J. & Albanes D. Prospective study of diet and pancreatic cancer in male smokers. American journal of epidemiology 155, 783–792 (2002). 11978580 10.1093/aje/155.9.783

[b32] Olsen G. W., Mandel J. S., Gibson R. W., Wattenberg L. W. & Schuman L. M. Nutrients and pancreatic cancer: a population-based case-control study. Cancer causes & control: CCC 2, 291–297 (1991). 1932541 10.1007/BF00051668

[b33] Ghadirian P., Simard A., Baillargeon J., Maisonneuve P. & Boyle P. Nutritional factors and pancreatic cancer in the francophone community in Montreal, Canada. International journal of cancer. Journal international du cancer 47, 1–6 (1991). 1845960 10.1002/ijc.2910470102

[b34] Zatonski W. . Nutritional factors and pancreatic cancer: a case-control study from south-west Poland. International journal of cancer. Journal international du cancer 48, 390–394 (1991). 2040534 10.1002/ijc.2910480314

[b35] Lin Y. . Nutritional factors and risk of pancreatic cancer: a population-based case-control study based on direct interview in Japan. Journal of gastroenterology 40, 297–301, doi: 10.1007/s00535-004-1537-0 (2005). 15830290

[b36] Zhou Y., Wang T., Meng Q. & Zhai S. Association of carotenoids with risk of gastric cancer: A meta-analysis. Clinical nutrition (Edinburgh, Scotland), doi: 10.1016/j.clnu.2015.02.003 (2015). 25726725

[b37] Wang Y., Cui R., Xiao Y., Fang J. & Xu Q. Effect of Carotene and Lycopene on the Risk of Prostate Cancer: A Systematic Review and Dose-Response Meta-Analysis of Observational Studies. PloS one 10, e0137427, doi: 10.1371/journal.pone.0137427 (2015). 26372549 PMC4570783

[b38] Zhang J. . Sequence variants in antioxidant defense and DNA repair genes, dietary antioxidants, and pancreatic cancer risk. International journal of molecular epidemiology and genetics 2, 236–244 (2011). 21915362 PMC3166151

[b39] Bueno de Mesquita H. B., Maisonneuve P., Runia S. & Moerman C. J. Intake of foods and nutrients and cancer of the exocrine pancreas: a population-based case-control study in The Netherlands. International journal of cancer. Journal international du cancer 48, 540–549 (1991). 1646177 10.1002/ijc.2910480411

[b40] Soler M., Chatenoud L., La Vecchia C., Franceschi S. & Negri E. Diet, alcohol, coffee and pancreatic cancer: final results from an Italian study. European journal of cancer prevention: the official journal of the European Cancer Prevention Organisation (ECP) 7, 455–460 (1998). 9926293 10.1097/00008469-199812000-00005

[b41] Jansen R. J. . Nutrients from fruit and vegetable consumption reduce the risk of pancreatic cancer. Journal of gastrointestinal cancer 44, 152–161, doi: 10.1007/s12029-012-9441-y (2013). 23620017 PMC3694591

[b42] Shibata A., Mack T. M., Paganini-Hill A., Ross R. K. & Henderson B. E. A prospective study of pancreatic cancer in the elderly. International journal of cancer. Journal international du cancer 58, 46–49 (1994). 8014014 10.1002/ijc.2910580109

[b43] Heinen M. M., Verhage B. A., Goldbohm R. A. & van den Brandt P. A. Intake of vegetables, fruits, carotenoids and vitamins C and E and pancreatic cancer risk in The Netherlands Cohort Study. International journal of cancer. Journal international du cancer 130, 147–158, doi: 10.1002/ijc.25989 (2012). 21328344

[b44] Han X. . Antioxidant intake and pancreatic cancer risk: the Vitamins and Lifestyle (VITAL) Study. Cancer 119, 1314–1320, doi: 10.1002/cncr.27936 (2013). 23280534 PMC3604041

[b45] Jeurnink S. M. . Plasma carotenoids, vitamin C, retinol and tocopherols levels and pancreatic cancer risk within the European Prospective Investigation into Cancer and Nutrition: a nested case-control study: plasma micronutrients and pancreatic cancer risk. International journal of cancer. Journal international du cancer 136, E665–676, doi: 10.1002/ijc.29175 (2015). 25175624

[b46] Munafo M. R. & Flint J. Meta-analysis of genetic association studies. Trends in genetics: TIG 20, 439–444, doi: 10.1016/j.tig.2004.06.014 (2004). 15313553

[b47] Roberts N. J. & Klein A. P. Genome-wide sequencing to identify the cause of hereditary cancer syndromes: with examples from familial pancreatic cancer. Cancer letters 340, 227–233, doi: 10.1016/j.canlet.2012.11.008 (2013). 23196058 PMC3652916

[b48] Wells G. A., D O’Connell B. S., Peterson J., Welch V., Losos M. & Tugwell P. The Newcastle-Ottawa Scale (NOS) for assessing the quality of nonrandomised studies in meta-analyses (2011).

[b49] Greenland S. Quantitative methods in the review of epidemiologic literature. Epidemiol Rev 9, 1–30 (1987). 3678409 10.1093/oxfordjournals.epirev.a036298

[b50] Egger M., Davey Smith G., Schneider M. & Minder C. Bias in meta-analysis detected by a simple, graphical test. BMJ (Clinical research ed.) 315, 629–634 (1997). 10.1136/bmj.315.7109.629 PMC2127453 9310563

